# Compromised Protein Prenylation as Pathogenic Mechanism in Mevalonate Kinase Deficiency

**DOI:** 10.3389/fimmu.2021.724991

**Published:** 2021-09-03

**Authors:** Frouwkje A. Politiek, Hans R. Waterham

**Affiliations:** Laboratory Genetic Metabolic Diseases, Amsterdam Gastroenterology, Endocrinology & Metabolism, Amsterdam UMC, University of Amsterdam, Amsterdam, Netherlands

**Keywords:** mevalonate kinase deficiency (MKD), protein prenylation, hyper IgD syndrome, mevalonic aciduria, isoprenoid biosynthesis

## Abstract

Mevalonate kinase deficiency (MKD) is an autoinflammatory metabolic disorder characterized by life-long recurring episodes of fever and inflammation, often without clear cause. MKD is caused by bi-allelic pathogenic variants in the *MVK* gene, resulting in a decreased activity of the encoded enzyme mevalonate kinase (MK). MK is an essential enzyme in the isoprenoid biosynthesis pathway, which generates both non-sterol and sterol isoprenoids. The inflammatory symptoms of patients with MKD point to a major role for isoprenoids in the regulation of the innate immune system. In particular a temporary shortage of the non-sterol isoprenoid geranylgeranyl pyrophosphate (GGPP) is increasingly linked with inflammation in MKD. The shortage of GGPP compromises protein prenylation, which is thought to be one of the main causes leading to the inflammatory episodes in MKD. In this review, we discuss current views and the state of knowledge of the pathogenetic mechanisms in MKD, with particular focus on the role of compromised protein prenylation.

## Introduction

Mevalonate kinase deficiency (MKD) is a rare autosomal recessive metabolic disorder, which is characterized by life-long recurring febrile and inflammatory episodes (1). The disorder is caused by bi-allelic pathogenic variants in the *MVK* gene, encoding mevalonate kinase (MK) and resulting in decreased activity of this enzyme (2–4). MK is a key enzyme in the biosynthesis of sterol and non-sterol isoprenoids and is the first enzyme to follow the well-known 3-hydroxy-3-methylglutaryl-CoA reductase (HMGR). The different isoprenoids play essential roles in a large variety of cellular processes, including membrane structure, steroid hormone and bile acid synthesis, cell growth, cell differentiation and protein prenylation (5–7).

Because of the characteristic recurrent inflammatory episodes accompanied with interleukin-1β (IL-1β) secretion, MKD also has been classified as an autoinflammatory disease. Autoinflammatory diseases comprise an increasing number of diseases that are characterized by recurrent and systemic inflammation caused by dysregulation of the innate immune system. These diseases are associated with increased production of pro-inflammatory cytokines. Well known autoinflammatory diseases are Familial Mediterranean Fever (FMF), TNF Receptor Associated Periodic Syndrome (TRAPS), systemic Juvenile Idiopathic Arthritis, Crohn’s disease and Behcet’s disease (8). In contrast to most autoinflammatory diseases, which are caused by pathogenic variants in genes encoding components directly involved in the innate immune system, the deficiency of MK in MKD implies a role for isoprenoids in the regulation of the innate immune response. Although the precise molecular mechanisms contributing to the inflammatory symptoms in MKD are not fully understood yet, increasing evidence suggests that altered protein prenylation is one of the main underlying causes. In this review we discuss the current state of knowledge on the pathogenesis of MKD with particular focus on the role of protein prenylation in the regulation of innate immunity.

## Clinical Aspects of MKD

The clinical presentation of patients with MKD may vary from recurrent inflammatory episodes associated with high fever, skin rash, abdominal pain, lymphadenopathy, splenomegaly and joint pain, previously diagnosed as Hyperimmunoglobulinemia D and Periodic Fever Syndrome (HIDS, MIM# 260920), to a severe multisystemic presentation with prenatal onset, also known as mevalonic aciduria (MA, MIM# 610377). In addition to similar recurrent inflammatory episodes as in the HIDS presentation, MA is associated with hepatosplenomegaly, dysmorphic features, ocular involvement, failure to thrive, psychomotor retardation and cerebellar ataxia. The severe MA phenotype can be fatal in childhood, whereas the HIDS phenotype generally does not have an effect on life expectancy (9). The recognition that HIDS and MA are not separate entities but represent the mild and severe end of the MKD spectrum only became clear when the genetic cause underlying HIDS was discovered in 1999 (2, 3). The genetic cause underlying MA had already been resolved in 1992 (10). Although the terms HIDS and MA are still used, it is now common to refer to these as the mild and the severe phenotypes of the MKD spectrum (11).

The first inflammatory episode in patients affected by the milder form of MKD (HIDS) typically occurs in the first year of life and is often triggered by childhood vaccinations. In addition to vaccinations, physical and emotional stress, and infections may evoke inflammatory episodes. However, in most cases, these inflammatory episodes seem to occur without a clear cause, a phenomenon also termed “sterile inflammation” (12–14). The inflammatory episodes usually last 3 to 7 days and almost half of the patients up till 10 years of age experience more than 12 episodes per year. Although this number decreases with age, still more than half of the patients aged 20 years and older experience 6 or more attacks per year. Between episodes, the majority of patients with the HIDS presentation are symptom free. However, the unpredictable course of MKD severely affects the lives of patients and family, such as causing educational delay, increasing unemployment and negatively affecting multiple aspects of quality of life (13). Patients with the severe MA presentation also experience episodes of recurrent inflammation, which in several cases has been fatal (9).

## Genotype-Phenotype Correlation in MKD

The clinical presentation of patients correlates well with the residual MK enzyme activity measured in cultured fibroblasts or blood cells of the patients (15, 16). Using an enzyme assay in which the conversion of radiolabeled mevalonate into its phosphorylated form is measured in cell lysates (17), the residual MK activity in fibroblasts from HIDS patients usually ranges from 4-10% when compared to the activity in controls (2, 3, 18, 19). The residual MK activity in fibroblasts from MA patients are below the detection level (<0.1%) (9, 15). Importantly, pathway flux analysis revealed that even in fibroblasts from MA patients, the flux through the pathway is not completely deficient, which implies that although the activity of MK is very low, it is not completely absent (20). The differences in residual MK activity are also reflected in the mevalonic acid levels in plasma and urine. In HIDS patients, the plasma and urinary levels of mevalonic acid are only slightly increased during an inflammatory episode, whereas in MA patients, these mevalonic acid levels are constitutively markedly elevated (2, 3, 9).

Currently, almost 200 different pathogenic variants in the *MVK* gene have been reported for MKD. The majority of these are missense and nonsense variants, but also deletions, insertions and splicing variants have been found (for current listing, see Human Gene Mutation Database at http://www.hgmd.cf.ac.uk). No patients with bi-allelic complete-loss-of-function variants have been reported, which is in line with the assumed essential role of MK in isoprenoid biosynthesis. The large majority of HIDS patients (>80%) is compound heterozygous or homozygous for the same c.1129G>A (p.V377I) variant in *MVK* (14, 15, 19), although the variant has also been reported in a few patients with severe MKD (19, 21). The second variant found in HIDS patients carrying the p.V377I variant, often causes the MA presentation when homozygous or in trans with another variant (e.g. p.H20P, p.I268T, or p.A334T) (14, 15). The p.V377I variant does not affect the catalytic activity of the mutant MK protein, but was shown to affect the correct folding, assembly and/or stability of the enzyme (16).

In general, there is a good correlation between the residual MK activity and MK protein levels in patient cells, as assessed by immunoblot analysis (15). Interestingly, the MK protein levels and the corresponding MK activity in cultured cells appear to be sensitive to temperature: culturing at 30°C results in increased MK levels and activity, while culturing at 39/40°C results in a clear decrease thereof. This phenomenon in particular has a major impact in cells with low residual MK levels and activity, such as the MVK-p.V377I cells; in these, elevated temperatures resulted in non-detectable MK protein levels and activities (16). As discussed below, this latter observation may explain in part the pathogenesis underlying MKD (16, 20).

## The Isoprenoid Biosynthesis Pathway

**Figure 1:**
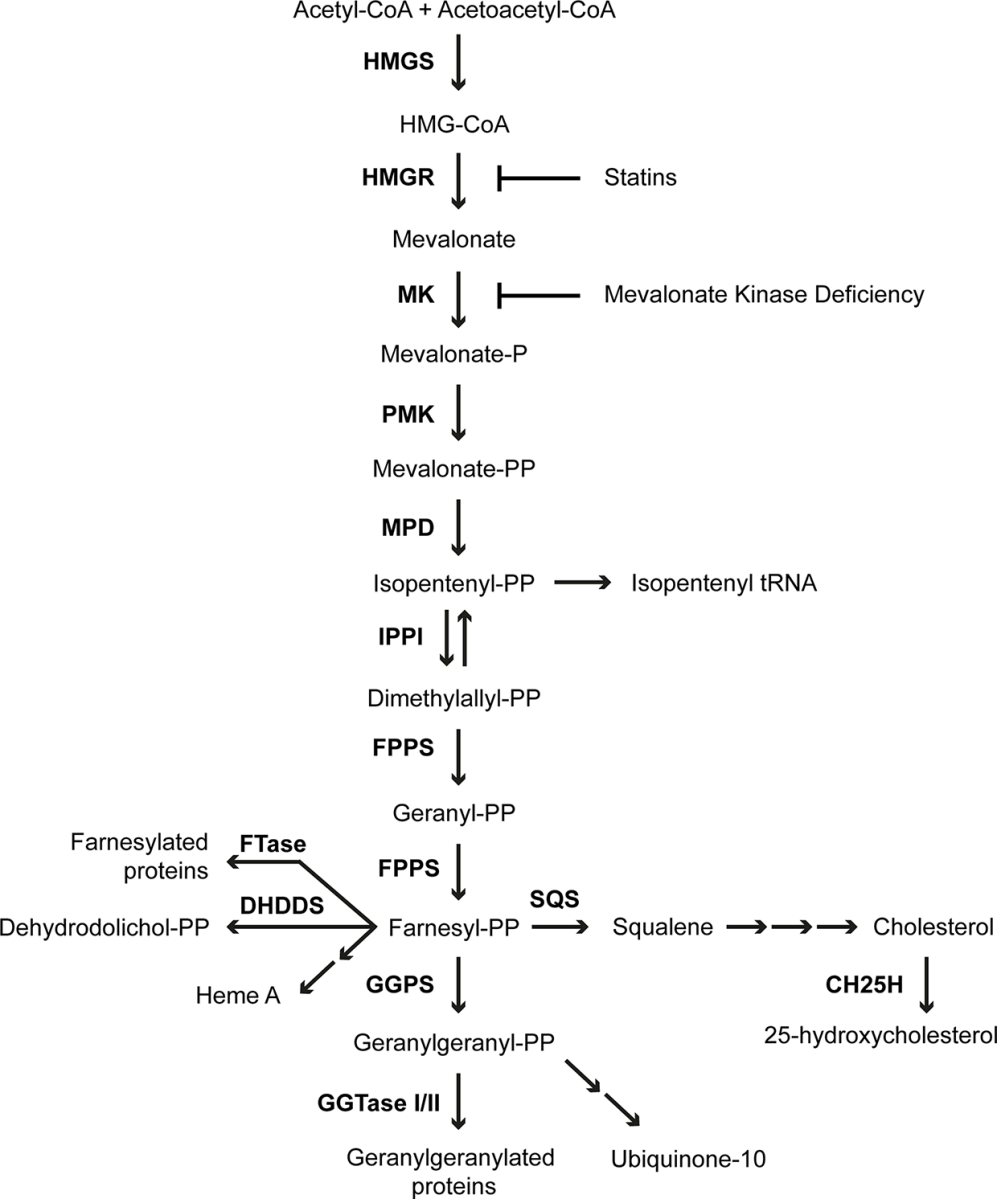
Isoprenoid biosynthesis pathway. The enzymes of the pathway are 3-hydroxy-3-methylglutaryl-CoA synthase (HMGS), 3-hydroxy-3-methylglutaryl-CoA reductase (HMGR), mevalonate kinase (MK), phosphomevalonate kinase (PMK), mevalonate pyrophosphate decarboxylase (MPD), isopentenyl pyrophosphate isomerase (IPPI), farnesyl pyrophosphate synthase (FPPS), farnesyltransferase (FTase), dehydrodolichyl diphosphate synthase (DHDDS), geranylgeranyl pyrophosphate synthase (GGPS), geranylgeranyltransferase (GGTase), squalene synthase (SQS) and cholesterol 25-hydroxylase (CH25H).

MK is an important enzyme in the isoprenoid biosynthesis pathway (**Figure 1**), which produces numerous sterol and non-sterol isoprenoids with essential cellular functions (7, 22). In the first step of isoprenoid biosynthesis, 3-hydroxy-3-methylglutaryl-CoA (HMG-CoA) is reduced to mevalonate by the rate-limiting and highly regulated enzyme HMGR. Next, MK phosphorylates mevalonate, yielding 5-phosphomevalonate. Subsequently, another phosphorylation step, catalyzed by phosphomevalonate kinase, yields 5-pyrophosphomevalonate. Decarboxylation of 5-pyrophosphomevalonate produces the 5-carbon isopentenyl pyrophosphate (IPP), which is the building block of all isoprenoids. In the following steps IPP is used to form the 15-carbon non-sterol isoprenoid farnesyl pyrophosphate (FPP). FPP is the branch point of the isoprenoid biosynthesis pathway from which the sterol and non-sterol branches of isoprenoid biosynthesis divert. The conversion of two molecules of FPP into squalene is the first committed step in the sterol biosynthesis branch of the pathway, which ultimately leads to the production of cholesterol. Cholesterol is an essential component of cellular membranes as well as a precursor for multiple molecules, including steroid hormones and bile acids. In the non-sterol branch of the isoprenoid biosynthesis pathway, FPP is used for the synthesis of heme A from protoheme as well as for the synthesis of dehydrodolichol pyrophosphate. Moreover, addition of another IPP to FPP generates the 20-carbon geranylgeranyl pyrophosphate (GGPP). Both FPP and GGPP are involved in protein prenylation, which is the posttranslational attachment of either an FPP or a GGPP moiety to target proteins, such as nuclear lamins, heterotrimeric G protein subunits and the Ras super family of small GTPases (23, 24). Prenylation is essential for correct localization and activation of target proteins. GGPP is also a precursor for the synthesis of ubiquinone-10.

## Regulation of the Isoprenoid Biosynthesis Pathway

The isoprenoid biosynthesis pathway is regulated at multiple levels to ensure the timely synthesis of isoprenoids when required and to prevent accumulation of possible toxic intermediates and end products, such as cholesterol (6, 22). The flux-controlling step of the pathway is at the level of HMGR, the activity of which can be controlled by gene transcription, efficiency of mRNA translation, rate of protein degradation and modulation of enzymatic activity. Transcriptional regulation of *HMGR* and other genes encoding enzymes in the pathway occurs *via* feedback regulation in response to sterol levels. This is mediated by the sterol regulatory element-binding protein 2 (SREBP-2). SREBP-2 and its relatives SREBP-1a and SREBP-1c are membrane-bound transcription factors involved in the regulation of cholesterol and fatty acid biosynthesis, lipogenesis and glucose metabolism. SREBP-2 regulates the transcription of the genes encoding the enzymes of the isoprenoid biosynthesis pathway as well as the LDL-receptor, which mediates the uptake of cholesterol as plasma lipoproteins (25, 26). SREBP-2 is synthesized as a large inactive precursor located in the ER membrane where it tightly associates with the SREBP-cleavage-activating protein (SCAP) escort protein. At high sterol concentrations, SCAP interacts strongly with one of the INSIG proteins, which causes the retention of the SREBP-2-SCAP complex in the ER. However, this interaction is weakened at low sterol concentrations, allowing the SREBP-2-SCAP complex to transfer to the Golgi apparatus. There, the N-terminal DNA binding/transcription activation domain is cleaved off from the C-terminal transmembrane/regulatory domain by the subsequent action of the two Golgi-bound proteases S1P and S2P. The N-terminal domain is then translocated to the nucleus where it activates transcription of the SREBP-2 target genes after binding to the sterol regulatory element (SRE) in the promotor regions of the genes (27).

Apart from transcriptional regulation by SREBP-2, HMGR is regulated at the translational and post-translational level. High sterol concentrations promote the binding of HMGR with cholesterol and one of the INSIG proteins, which leads to ubiquitination and subsequent proteasomal degradation of HMGR. This ubiquitination can be stimulated by lanosterol, the first sterol intermediate in cholesterol biosynthesis, as well as by oxygenated derivatives thereof and cholesterol-derived oxysterols such as 25-hydroxysterols (28–32). This process is accelerated in the presence of non-sterol isoprenoids (30, 33–38). HMGR levels are also regulated at the translational level by increased levels of non-sterol isoprenoids, which cause a reduction in the translation rate of HMGR mRNA (39).

## Transient Compromised Isoprenoid Biosynthesis in MKD

Because MK is an early enzyme in the isoprenoid biosynthesis pathway, its deficiency in MKD in principle would be expected to compromise the biosynthesis of all isoprenoids. In patients with the severe MA presentation, who have very low MK activities, this is indeed reflected in lowered plasma ubiquinone-10 levels and, in some patients, in slightly reduced plasma cholesterol levels (9, 40, 41). However, the absence of lactic acidosis suggests that ubiquinone-10 levels are sufficient to maintain mitochondrial electron transport activity (9), while the *in vitro* levels of ubiquinone-10 in fibroblasts from MA patients are comparable to controls (42). In patients with the HIDS presentation, who have low residual MK activities, serum cholesterol levels are normal (43). Moreover, in cultured skin fibroblasts from HIDS patients, the flux through the isoprenoid biosynthesis pathway is similar to the flux in control fibroblasts under normal culture conditions. Earlier work by Houten et al. has revealed that this is due to an increased HMGR activity in these cells, which leads to increased levels of intracellular mevalonate assuring sufficient flux through the isoprenoid biosynthesis pathway and, consequently, the synthesis of isoprenoids (20). A similar increase in HMGR activity was also found in peripheral blood mononuclear cells (PBMCs) from MKD patients. Interestingly, the MK and HMGR activities showed a clear inversed correlation when measured in PBMCs drawn from patients with the HIDS presentation during or between fever episodes: during fever, the MK activity is decreased and the HGMR activity increased when compared to the activities between a fever episode (16).

These observations combined with what is known about the regulation of the isoprenoid biosynthesis pathway, have led to the following hypothesis for the pathogenesis underlying the episodic inflammatory symptoms in MKD (16, 20): under normal conditions, the increased HMGR activity in cells from MKD patients results in an elevated mevalonate level, which compensates for the decreased MK activity in the cells and assures a normal flux through the pathway. However, due to the temperature-sensitive nature of MK, an increase in temperature, e.g. during fever or due to exercise, stress or infections, will cause a rapid further decrease of the MK activity in these cells, which leads to a temporary decrease or block in the pathway flux and, accordingly, in the synthesis of isoprenoids. This in particular affects isoprenoids with a high-turnover rate among which isoprenoids that are involved in or required for the regulation of the onset and/or dampening of an inflammatory response, which thus causes the inflammatory phenotype of MKD. Several studies, discussed in more detail below, have shown that in particular the temporary shortage of the non-sterol isoprenoid GGPP is the main factor causing the increased HMGR activity and the inflammatory phenotype in MKD. An important first indication for this phenomenon came from patient fibroblast studies in which the effect of supplementation of GGPP or 25-OH cholesterol on the increased HMGR activities in MKD and familial hypercholesterolemia (FHC) cells were analyzed. While the HMGR activity in FHC cells was more responsive to 25-OH cholesterol, the activity in MKD cells was more responsive to GGPP (20).

## Immunological Characteristics of MKD

MKD is a member of the group of autoinflammatory diseases, which are all caused by defects in genes encoding components involved in the innate immune response. In contrast to autoimmune diseases, in which the adaptive immune system is affected and autoantibodies are produced, the autoinflammatory diseases have a defect in the innate immune system, resulting in a dysregulated pro-inflammatory cytokine response. The inflammatory phenotype of MKD highlights a role for isoprenoids in the regulation of innate immunity. Within the group of autoinflammatory diseases, MKD belongs to the periodic fever syndromes, which are caused by a dysregulation of the inflammasome-mediated release of the pro-inflammatory cytokine IL-1β (44). Inflammasomes are key players in the innate immune response and crucial for the release of active IL-1β. These multi-protein complexes are assembled following the recognition of pathogen- or damage-associated molecular patterns. Upon assembly they promote activation of caspase-1, resulting in the proteolytic cleavage of the non-active pro-IL-1β into its active form IL-1β (45). IL-1β is an early component of the pro-inflammatory cytokine pathway and a key player in the inflammatory presentation of MKD. Therefore blocking the receptor of IL-1β with biological agents is an important treatment option in patients with MKD (46).

In addition to increased IL-1β release, the inflammatory episodes of MKD patients are characterized by an acute phase response, reflected by elevated erythrocyte sedimentation rates (ESR), leukocytosis and elevated serum levels of C-reactive protein (CRP) and serum amyloid A (SAA). Levels of the pro-inflammatory cytokines tumor necrosis factor-α (TNF-α) and interleukin-6 (IL-6) were also found to be elevated (12, 47–49). Initially, constantly raised IgD levels were also thought to be a hallmark of HIDS. However, after the finding that HIDS, as MA, is caused by pathogenic variants in *MVK*, it has become apparent that not all patients with genetically confirmed MKD have elevated IgD levels (13, 50). Furthermore, elevated IgD levels have also been reported in other diseases. Finally, there is no correlation between IgD levels and disease severity (12), rendering it unlikely that IgD plays a major role in the inflammatory presentation.

Innate immune cells of MKD patients have an increased pro-inflammatory phenotype. PBMCs of the patients release higher amounts of pro-inflammatory cytokines, mainly IL-1β, TNF-α and IL-6, in stimulated conditions as well as spontaneously (48, 51–53). As will be discussed below, in particular the temporary lack of newly synthesized GGPP was found to be linked to the enhanced inflammatory responses seen in MKD.

More recently, mevalonate was shown to induce *ex vivo* trained immunity in blood monocytes (54). Trained immunity is the long-term non-specific memory of the innate immune system and is mediated by epigenetic and metabolic reprogramming leading to an enhanced non-specific immune response of innate immune cells upon their next stimulation (55). Although the mevalonate accumulation in patients with the HIDS phenotype is minimal, monocytes of these patients indeed showed a trained immunity phenotype characterized by epigenetic changes, increased expression of glycolytic genes and increased cytokine production (54). Thus, in addition to a temporary shortage of GGPP, also the accumulation of mevalonate appears to contribute to the hyper-inflammatory phenotype seen in MKD patients.

## Consequences of Disturbed Isoprenoid Biosynthesis on Prenylation of Small Rho GTPases

Multiple studies have shown that the temporary shortage of in particular GGPP affects protein prenylation in MKD and that this is one of the important causes of inflammation. The majority of prenylated proteins are so-called CAAX proteins. The C-terminal CAAX motif is recognized by protein farnesyltransferase (FTase) and protein geranylgeranyltransferase I (GGTase I), which catalyze the covalent attachment of the 15-carbon FPP or the 20-carbon GGPP to the cysteine residue of the CAAX motif. Besides the cysteine, the CAAX motif contains two aliphatic amino acids (A) while the X can be one of a variety of amino acids. The X amino acid determines whether FTase or GGTase I will bind to the target CAAX protein and thus whether a protein will be farnesylated or geranylgeranylated (23).

**Figure 2:**
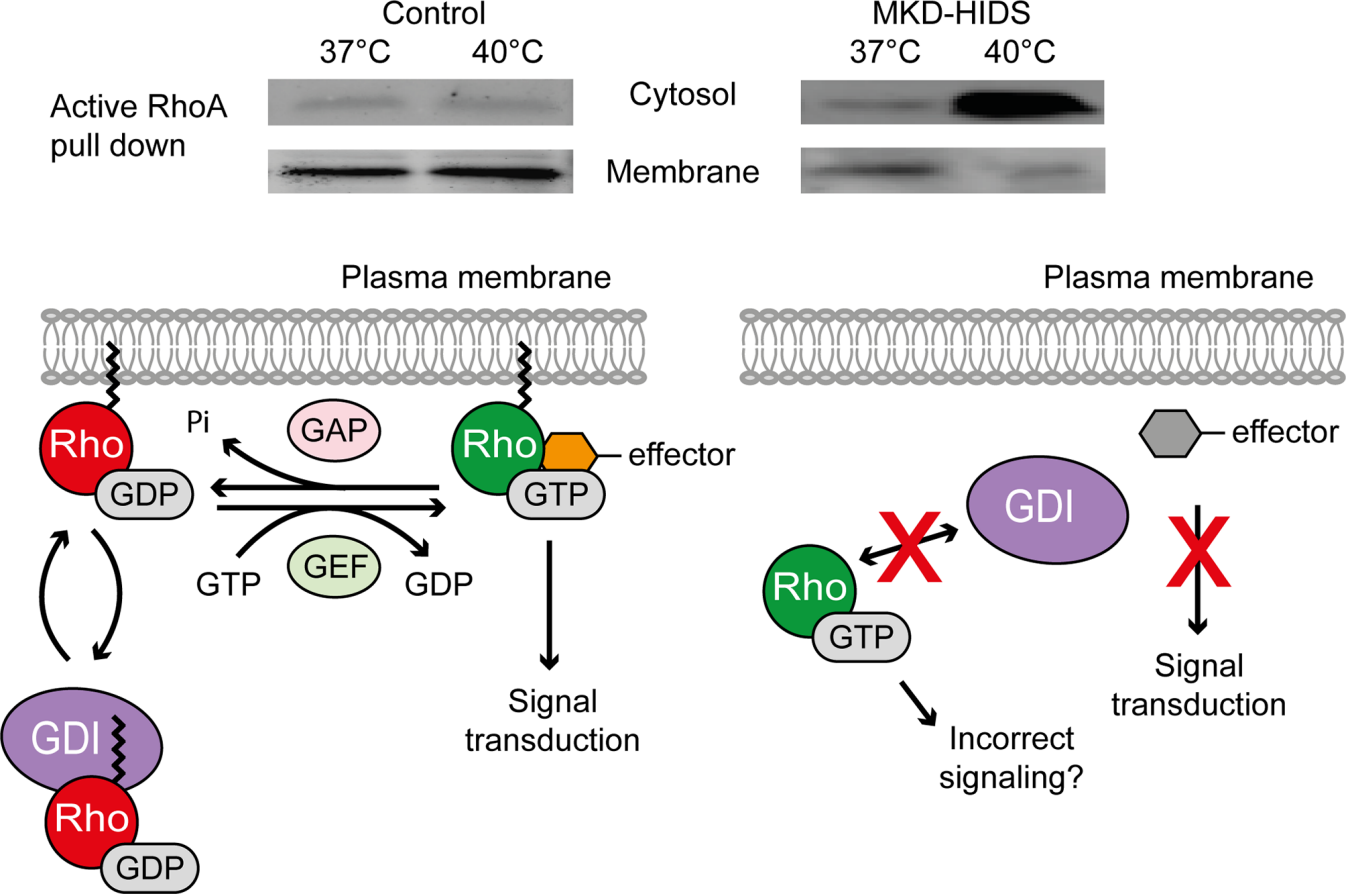
Effect of temperature on the activation and localization of the small GTPase RhoA in MKD. Switching cells of MKD patients from 37°C to 40°C leads to GGPP depletion affecting protein geranylgeranylation and causing ectopic activation of the small GTPase RhoA. Guanine nucleotide dissociation inhibitor (GDI), guanine nucleotide exchange factor (GEF), GTPase activating protein (GAP).

The largest group of prenylated proteins are the small GTPases (24). Small GTPases are highly dependent on prenylation for their correct functioning (**Figure 2**). The hydrophobic prenyl group enables protein-protein interactions, but also allows GTPases to attach to cellular membranes. Attached to the membrane they can interact with other proteins, including kinases and adaptor proteins, to induce downstream signaling pathways (23). Most GTPases act as molecular switches in a wide variety of cellular processes, including cytoskeletal function, cellular adhesion, vesicle trafficking and cell cycle. By cycling between an active GTP-bound and an inactive GDP-bound state, signaling pathways will be switched on or off. This GDP-GTP cycling is regulated by guanine nucleotide exchange factors (GEFs), GTPase-activating proteins (GAPs) and GDP dissociation inhibitors (GDIs). GEFs catalyze the exchange of a GDP for a GTP, which activates the GTPases, while GAPs stimulate the GTPases to hydrolyze GTP to GDP, which inactivates the GTPases. The GDIs bind to the C-terminal prenyl group of GDP-bound GTPases in the cytosol, thereby maintaining their inactive state (5, 24).

The instant lack of non-sterol isoprenoids in MKD, in particular GGPP, caused by the (temporary) block of their synthesis was shown to result in the ectopic activation of small Rho GTPases in cells and a strong IL-1β release by monocytic cells (20, 51, 52, 56). Different studies have focused on the consequences of the reduced isoprenoid biosynthesis on the small Rho GTPases RhoA and Rac1, which require geranylgeranylation for their location in membranes and subsequent activation. In skin fibroblasts from MKD patients, decreased membrane-bound and increased soluble levels of activated RhoA and Rac1 were observed at lower concentrations of simvastatin, an inhibitor of HMGR, than in control fibroblasts (20, 56). This shows that the flux in the isoprenoid biosynthesis pathway in MKD cells is more sensitive to disturbances, e.g. by low concentrations of simvastatin. This results in the depletion of GGPP, as a consequence of which the GTPases will not become geranylgeranylated and thus neither can interact with the GDIs nor can be targeted to the membrane. The result of this is that the GTPases become active in the cytosol. A similar effect on the location and activation of RhoA and Rac1 is seen when MKD cells are switched to 40°C, which, as mentioned above, causes a rapid decrease in MK protein levels and residual activity, causing a shortage of newly synthesized GGPP (**Figure 2**).

Because fibroblasts do not have an innate immunological phenotype, they are not suitable to study whether the ectopic activation of RhoA and Rac1 leads to the induction and/or loss of suppression of inflammatory signaling pathways. As an alternative, the human monocytic cell line THP-1 has been treated with simvastatin to mimic the shortage of non-sterol isoprenoids seen in MKD. In contrast to the findings in fibroblasts from MKD patients, where both the activated RhoA and Rac1 levels increased upon simvastatin treatment, the treatment of THP-1 cells caused a decrease in the levels of activated RhoA, whereas the levels of activated Rac1 increased (57, 58). Moreover, inhibition of RhoA in THP-1 cells resulted in increased Rac1 activation and IL-1β mRNA levels (58). The increased Rac1 activity was shown to enhance IL-1β release *via* activation of caspase-1 through the Rac1-PI3K-PKB pathway (57). The effect of ectopic activation of RhoA and Rac1 was also studied in THP-1 cells transiently expressing RhoA or Rac1 lacking the C-terminal CAAX motif, which prevents their prenylation and thus mimics the situation in MKD cells. Although to a lesser extent when compared to simvastatin treatment, expression of CAAX-deficient RhoA and Rac1 resulted in increased levels of phosphorylated PKB (58). Decreased LPS-induced IL-1β release by PBMCs of MKD patients treated with an Rac1 inhibitor suggested a role of Rac1 in increasing IL-1β secretion (57).

Earlier studies with PBMCs from MKD patients showed a marked increase in LPS-stimulated secretion of IL-1β when compared to control PBMCs. This increased IL-1β secretion could be suppressed in part by supplementing FPP and fully by supplementing GGPP prior to the LPS stimulation (51, 52). Moreover, inhibition of the geranylgeranyl transferase GGTase I in control PBMCs resulted in increased IL-1β release upon LPS stimulation, in contrast to no response upon inhibition of the farnesyl transferase FTase. This confirms that deficient protein geranylgeranylation and not farnesylation contributes to the inflammatory phenotype (52).

In line with the above findings, Rac1 is responsible for the activation of the innate immune response in mice with GGTase I-deficient macrophages (59). In these mice, the GGTase I deficiency in macrophages leads to an inflammatory phenotype, characterized by enhanced release of pro-inflammatory cytokines, activation of inflammatory signaling pathways and severe joint inflammation, thereby resembling rheumatoid arthritis. In the GGTase I-deficient macrophages the GTP-bound levels of Rac1, RhoA and Cdc42 were increased. Although the morphology of the GGTase I-deficient macrophages was found to be altered, migration and phagocytosis were similar as in control macrophages. Inhibition of TNF-α was found to reduce synovitis *in vivo*, while inhibition of Rac1 in the GGTase I-deficient macrophages reduced LPS-stimulated IL-1β and TNF-α release (60). Furthermore, a heterozygous deletion of Rac1 in the GGTase I-deficient macrophages reversed the inflammation in this mouse model, whereas a heterozygous deletion of RhoA and Cdc42 did not. Non-prenylated Rac1 was found to have an increased interaction with the adaptor protein Ras GTPase-activating-like protein 1 (Iqgap1), which was shown to lead to pro-inflammatory signaling in GGTase I-deficient macrophages, strongly suggesting that under normal circumstances prenylation of Rac1 prevents the activation of inflammatory signaling pathways (59). So far, the role of Rac1-Iqgap1 or other Rac1-effector interactions in MKD have not been studied.

Recently, also the altered subcellular localization of the small Rho GTPase CDC42 has been linked to autoinflammation in patients. Lam et al. described a novel haematological and autoinflammatory syndrome caused by altered localization and functioning of CDC42. CDC42 is involved in multiple cellular processes, including adhesion, polarization, cell cycle, migration and regulation of the cytoskeleton. In four unrelated patients, the same *de novo* missense variant in the *CDC42* gene (p.R186C) resulted in a dominant multisystem syndrome characterized by neonatal onset cytopenia with dyshematopoiesis, autoinflammation, rash, and episodes of hemaphagocytic lymphohistiocytosis (NOCARH syndrome) (61). Variants in *CDC42* already had been reported to cause a range of neurodevelopmental phenotypes and were found to alter CDC42 functioning by affecting the GDP-GTP cycling and/or by affecting the interaction of CDC42 with regulatory and effector proteins (62). In contrast to these patients, however, the patients with the p.R186C variant had no neurodevelopmental symptoms. Functional analysis revealed that the p.R186C variant does not affect GDP-GTP cycling, but results in retention of CDC42 in the Golgi apparatus (61) due to the palmitoylation of the mutated p.R186C (63). More recently, additional C-terminal variants in *CDC42* were found in patients presenting with CDC42-related autoinflammation, including a p.C188Y variant. This variant affects the cysteine that is normally prenylated and thus is predicted to lead to non-prenylated CDC42 proteins, although this had not been studied. Taken together, these findings link an altered subcellular localization of CDC42, either due to aberrant palmitoylation (p.R186C) or to impaired prenylation (p.C188Y), to the development of autoinflammatory symptoms (63, 64). This is in line with the postulated link between inflammation and an altered localization of GTPases in MKD.

In addition to the small Rho GTPases, which are geranylgeranylated by GGTase I, the prenylation of the Rab GTPases is also affected in MKD. Rab GTPases are exclusively geranylgeranylated by a third protein prenyltransferase named GGTase II or Rab GGTase. They lack a conserved motif like the CAAX motif, but are able to interact with Rab escort protein (REP) to form a complex. The Rab-REP complex is recognized by Rab GGTase, which geranylgeranylates most Rab GTPases at two C-terminal cysteine residues (65). As with the Rho GTPases, the deficiency of MK leads to increased levels of non-prenylated Rab GTPases in patient-derived PBMCs and in lymphoid cells from MKD patients cultured at elevated temperature (40°C) (66, 67). Moreover, in three patients, the residual MK activity negatively correlated with accumulation of non-prenylated Rab GTPases (66). However, in THP-1 cells the inhibition of GGTase II by NE10790 had no effect on LPS-stimulated IL-1β release, whereas the inhibition of GGTase I by GGTI-298 did enhance IL-1β release. Incubation of control PBMCs with NE10790 also did not affect IL-1β release, whereas incubation with GGTI-298 resulted in the release of IL-1β. This indicates that the loss of prenylation of Rab GTPases in MKD does not lead to IL-1β release, whereas, in agreement with previous data (52), loss of prenylation of the GTPases that are prenylated by GGTase I, including Rho, Rac and Rap GTPases, does contribute to IL-1β release in MKD (67). Despite this finding, Rab GTPases are known to be key players in membrane trafficking and immune cell functions as endocytosis, phagocytosis and cytokine release (68). Future research should elucidate whether and, if so, how altered prenylation of Rab GTPases contributes to the pathology of MKD.

## Impaired Geranylgeranylation of GTPases and Inflammasome Activation

So far, two studies have linked the impaired geranylgeranylation of small GTPases in MKD to activation of the pyrin inflammasome. Activation of the pyrin inflammasome leads to the activation of caspase-1 and subsequent release of active IL-1β and IL-18 (69). The signal for pyrin inflammasome activation had remained unknown for a long time. In 2014, however, it was discovered that pyrin can sense pathogens in an indirect manner, namely *via* the downstream effects of pathogen-induced loss of Rho GTPase activity (70). Following this discovery, impaired geranylgeranylation of GTPases was also reported to lead to the activation of the pyrin inflammasome (71, 72). Akula et al. showed that geranylgeranylation is required for the interaction between the small Ras GTPase Kras and the p110δ subunit of PI3K. The Kras-p110δ interaction is essential for the Toll-like receptor (TLR)-induced activation of PI3K and downstream signaling pathways. Lack of protein geranylgeranylation led to impaired PI3K signaling, which resulted in a hyper-inflammatory response, including pyrin inflammasome activation (71). Park et al. showed that the activity of the small GTPase RhoA suppresses activation of the pyrin inflammasome. In short, they found that RhoA signaling activates protein kinase N1 and N2 (PKN), leading to the phosphorylation of pyrin. When phosphorylated, pyrin binds to so-called 14-3-3 proteins, which suppresses the activation of the pyrin inflammasome. Decreased GGPP synthesis resulted in reduced RhoA signaling and subsequent activation of the pyrin inflammasome. The role of the pyrin inflammasome in the pathogenesis of MKD was confirmed in PBMCs from HIDS patients, in which pharmacological activation of PKN, leading to the downstream inhibition of the pyrin inflammasome, reduced IL-1β release (72). Of note, the authors also studied the anti-inflammatory effects of colchicine in PBMCs of MKD patients. One of the mechanisms underlying the anti-inflammatory effects of colchicine is the inhibition of microtubule polymerization, which leads to the release of GEF-H1 and subsequent activation of RhoA. Activated RhoA can then activate protein kinase N1 and N2, thereby inhibiting pyrin inflammasome activation (72, 73). However, in PBMCs of MKD patients, colchicine did not inhibit pyrin inflammasome activation. The authors stated that this is most likely due to the presence of non-geranylgeranylated RhoA, that cannot attach to the membrane and therefore cannot be activated by colchicine (72). This may explain why clinically, colchicine treatment was found not to be effective in MKD (14).

In addition to the pyrin inflammasome, activation of the NLRP3 inflammasome has also been linked to IL-1β release in MKD (74). Of all inflammasomes, the NLRP3 inflammasome is the best studied and the most sensitive to metabolic changes (45). Loss of prenylation in simvastatin-treated THP-1 cells enhanced LPS-induced inflammation in an NLRP3-dependent manner. In contrast to the above finding that altered prenylation can lead to activation of the pyrin inflammasome, Skinner et al. found that IL-1β release is independent of the pyrin inflammasome. Furthermore, stimulation with LPS and nigericin of PBMCs from an MKD patient, which triggers NLRP3 activation, resulted in increased IL-1β release compared to the patients’ parents. Subsequent inhibition of the NLRP3 inflammasome with MCC950 completely inhibited IL-1β release (74). Another study reported that knock-down of *MVK* in a murine microglial cell line (BV-2) did not affect protein expression of NLRP3 (75). However, it should be noted that the knock-down of *MVK* led to a 40% decrease of MK protein levels, whereas MKD patients have significantly lower levels of MK, which makes it plausible that these findings do not reflect the pathologic processes of MKD. In an additional experiment BV-2 cells were incubated with lovastatin and LPS. Treatment with lovastatin alone increased MK protein levels, but did not affect NLRP3 protein levels, whereas the combined treatment with lovastatin and LPS increased both MK and NLPR3 protein levels (75). Also, incubation of PBMCs from healthy controls with both the farnesyl pyrophosphate synthase inhibitor alendronate and LPS caused a marked increase in IL-1β release and mRNA expression levels of *NLRP3*. In addition, the expression of *NLRP3* was found to be increased in PBMCs of 2 patients with MKD (76).

Taken together, the precise role and the mechanisms leading to the activation of the NLRP3 inflammasome in MKD are still unclear. The complexity of the regulation and signaling of the different GTPases makes it challenging to precisely identify which GTPases are affected by reduced prenylation in MKD, and how this affects different signaling pathways leading to inflammation in different cell types and *in vivo.* That being said, the finding of a direct link between altered prenylation of the GTPases RhoA and Kras (71, 72) and activation of the pyrin inflammasome in MKD is in full agreement with the early postulation that loss of prenylation is one of the important causes leading to inflammation in MKD (16, 20).

## Mitochondrial Dysfunction and Impaired Autophagy in MKD

Other processes that have been linked to compromised protein prenylation in MKD are mitochondrial function and autophagy. Ubiquinone-10 and heme A, both products of the isoprenoid biosynthesis pathway, function in the mitochondrial respiratory chain. In addition, GTPases are involved in mitochondrial fission and fusion, as well as in the regulation of autophagy. Accordingly, blockage of the isoprenoid biosynthesis pathway is not only associated with altered prenylation, but also with defective autophagy, mitochondrial dysfunction and apoptosis, all of which could potentially set off a chain of events leading to activation of the inflammasome and subsequently inflammation [for reviews see (77, 78)].

Multiple studies have focused on mitochondria and autophagy in *in vitro* MKD models. In brief, inhibition of isoprenoid synthesis by simvastatin resulted in impaired autophagy in THP-1 cells, subsequently causing an accumulation of damaged mitochondria and increased IL-1β release. Incubation with GGPP rescued the phenotype induced by simvastatin, underlining the involvement of protein geranylgeranylation in autophagy. Importantly, autophagy was also found to be defective in PBMCs of MKD patients. Moreover, induction of autophagy decreased the LPS-induced IL-1β release in PBMCs from healthy controls, whereas induction of autophagy had no significant effect on LPS-induced IL-1β release in PBMCs from MKD patients (79). In line with this, transient overexpression of *MVK* variants I268T and N301T in neuronal SH-SY5Y cells was found to increase cytosolic RhoA levels, impair autophagy and increase apoptosis (80). In another study, however, the transient expression of CAAX-deficient RhoA in THP-1 cells did not affect mitochondrial membrane potential and autophagy, although it resulted in mitochondrial elongation (58). A study looking at apoptosis of lymphocytes revealed decreased apoptosis of cells from HIDS patients, but normal apoptosis of lymphocytes of patients with FMF or TRAPS, two other autoinflammatory diseases (81). However, in other studies and cell-types, a reduced apoptosis has been reported in TRAPS (82, 83). Although the reduced apoptosis of lymphocytes from HIDS patients had been suggested to (also) play a role in the pathogenesis of HIDS (81), it remains unclear to which extent this affects the inflammatory symptoms in MKD.

Finally, 25-hydroxycholesterol (25-HC), a metabolite formed from cholesterol, was studied with regard to its potential to reverse/prevent inflammation and restore autophagy and apoptosis. Cholesterol-derived oxysterols, including 25-HC, function as bioactive lipids in the immune system and are increasingly studied for their role in inflammatory signaling pathways (84). Mice deficient for the gene encoding cholesterol 25-hydroxylase *(Ch25h)*, the enzyme producing 25-HC, showed enhanced LPS-stimulated release of IL-1β, IL-18 and IL-1α. Combined results of the Ch25h-deficient mice and macrophages showed that 25-HC plays an essential role in the suppression of IL-1 cytokine release downstream of type I IFN signaling (85). In glioblastoma cells (U87-MG), 25-HC indeed inhibited the lovastatin-induced IL-1β release. However, incubation with 25-HC did not restore protein prenylation, autophagy and apoptosis in this model (86). Although this has not been studied in MKD patients, a deficiency of MK is expected to lead to reduced levels of 25-HC. Further study remains to determine whether 25-HC levels are relevant in MKD.

## Isoprenoids and the Adaptive Immune Response

As discussed so far, most studies on MKD have focused on the consequences of a defective isoprenoid biosynthesis for the regulation of the innate immune system, the dysregulation of which is assumed to be responsible for the inflammation. Recently, however, the isoprenoid biosynthesis pathway, including GGPP, was also found to be required for IL-10 release by B cells, which are cells of the adaptive immune system. IL-10 is an important anti-inflammatory cytokine, which can be released by immune cells of both the innate and the adaptive immune system. The main function of IL-10 is to suppress a pro-inflammatory immune response, thereby preventing uncontrolled inflammation and damage to the host (87). In a recent study it was found that IL-10 release by B cells is controlled in a GGPP-dependent manner *via* the activation of PI3Kδ-AKT signaling and subsequent downstream inhibition of GSK3 (88). Moreover, in addition to GGPP, GGTase I activity was found to be required for IL-10 release, strongly suggesting that geranylgeranylated proteins are not only involved in secretion of the pro-inflammatory IL-1β, but also in the release of the anti-inflammatory IL-10. In line with this, B cells from MKD patients showed a defect in IL-10 production, which could be restored by supplementation with GGPP and inhibition of GSK3. Moreover, B cells of MKD patients had a reduced capacity to inhibit IFN-γ release by T cells. In contrast to the B cells, the cytokine levels in T cells, including IL-10, were similar between MKD patients and controls. In another study, inhibition of isoprenoid biosynthesis in IFN-γ producing T cells with atorvastatin or 25-HC prevented the cells to switch to an IL-10 expressing phenotype, but this was found to be independent of prenylation (89). In this study, the inhibition of the isoprenoid biosynthesis pathway was linked to a decreased expression of c-Maf, a transcription factor for IL-10.

It is unclear whether these findings can be translated also to cells of the innate immune system, which also can produce IL-10. However, these findings indicate that a disturbed isoprenoid biosynthesis and in particular a shortage of GGPP may disturb the balance between pro- and anti-inflammatory cytokines and thus contributes to the pro-inflammatory phenotype of MKD.

## Treatment Options for MKD

Current treatment of MKD patients is mainly focused on suppression of inflammation [for recent reviews see (46, 90)]. Mildly affected patients might benefit from suppression of inflammation by paracetamol, non-steroidal anti-inflammatory drugs and/or corticosteroids. Although these therapies are often used, evidence for the efficacy of these therapies in the treatment of MKD is limited (90, 91).

So far, anti-IL-1 therapies have been the most effective in the treatment of MKD, underlining the central role of IL-1β in MKD. In fact, administration of canakinumab, a human monoclonal antibody against IL-1β, is currently the only FDA- and EMA-approved treatment for MKD. This approval was based on the outcome of the CLUSTER study, which showed that canakinumab controlled and prevented the inflammatory episodes in the majority of the 72 included MKD patients (92). However, compared to patients with FMF and TRAPS, two other autoinflammatory diseases associated with increased IL-1β release, patients with MKD required a higher dose of canakinumab to efficiently control the inflammatory episodes. Canakinumab administration was found to be an effective and safe therapy for MKD in multiple studies with smaller study populations (14, 93–95). A major advantage of canakinumab is that, due to its long half-life, it only needs to be injected approximately every 4 to 8 weeks (46).

Another anti-IL-1 therapy used in the treatment of MKD patients is the administration of anakinra, a recombinant human IL-1 receptor antagonist (46, 90). The continuous use of anakinra was found to be partially effective in the majority of patients (14, 94, 95), while the on-demand use of anakinra was shown to reduce the severity and the duration of the inflammatory episodes. Because anakinra needs to be injected daily and is less effective than canakinumab, the latter is preferred, particularly in pediatric patients. However, treatment outcomes differ per patient, and the relatively low costs of anakinra treatment compared to canakinumab may influence the treatment choice (90, 96).

An interesting potential strategy to prevent the increased IL-1β release in MKD could be the use of NLRP3 inhibitors. So far, NLRP3 inhibitors have only been studied *ex vivo* in cells of one MKD patient (74) and were found to prevent LPS-induced IL-1β release, thus suggesting that NLRP3 inflammasome activation is involved in the pathogenesis of MKD. Since NLRP3 inflammasome activation has been associated with many diseases, there is a great interest in developing such inhibitors, some of which are currently studied in clinical trials (97).

When anti-IL-1 treatment is not effective, biologicals targeting TNF-α and IL-6 might be beneficial. Anti-TNF treatment is given in the form of etanercept, a recombinant human TNF receptor p75-Fc fusion protein. The effectiveness of etanercept varies among studies, leading to a beneficial response in approximately of 60% of reported patients (14, 19). Evidence for the effectiveness of anti-IL-6 therapy in MKD is mainly derived from a few case studies in which tocilizumab, a monoclonal antibody against the IL-6 receptor, resulted in the remission of inflammatory episodes in MKD patients (98–101). Another study reported four patients, who discontinued tocilizumab treatment because of lack of efficiency (102).

Eight severely affected MA patients who did not respond to anti-inflammatory therapies underwent stem cell transplantation. In four patients, the stem cell transplantation was effective and patients remained in remission in the follow-up period ranging from 15 months to 5 years (103–106). One patient died due to the consequences of sepsis several months after a bone marrow transplantation (107). Another patient remained in remission for 18 months, after which symptoms reoccurred, although they were less severe, suggesting that the transplantation led to a switch from the MA towards the HIDS phenotype of MKD (108). Recently, two MA patients were described who received an α/β T-cell and B-cell depleted stem cell transplantation that led to remission of inflammation in both patients. However, urinary mevalonic acid levels remained increased, probably due to MK deficiency still being present in other tissues (109).

Treatment options aimed at increasing the residual MK activity and/or bypassing the enzyme defect in the isoprenoid biosynthesis pathway to assure the synthesis of GGPP in principle could be beneficial in controlling or even preventing the inflammatory episodes. In this respect it is important to realize that parents or siblings of MKD patients, who are carriers of only one pathogenic variant in the *MVK* gene, are completely free of symptoms while showing MK enzyme activities ranging from 20-45% (3). Thus, compared to the 2-10% residual MK activity in HIDS patients, an increase of only 10-15% in MK activity should be sufficient to make HIDS patients less sensitive if not free of developing spontaneous inflammation. So far, however, interference with isoprenoid biosynthesis has not been studied in MKD patients, with the exception of statins. The rationale of treating HIDS patients with statins was that it would reduce the levels of mevalonate, assumed to be toxic in this study, as well as that it could lead to an increase in residual MK activity *via* SREBP-2 activated *MVK* gene transcription. Although the treatment resulted in minimal improvement in some patients, it was not effective in most patients and in patients with the severe MA presentation of MKD it even worsened the symptoms (9, 14, 110).

Several *in vitro* cell studies have shown that manipulation of the isoprenoid biosynthesis pathway in MKD cells to stimulate the synthesis of GGPP may be beneficial. This includes supplementation of intermediate isoprenoids or enzyme inhibitors to fibroblasts and PBMCs of patients. As already mentioned in this review, supplementation of MKD fibroblasts or PBMCs with the intermediate isoprenoids mevalonate, FPP, GGPP, and farnesol and geranylgeraniol (which both are converted intracellularly to FPP and GGPP), resulted in reduction of the increased HMGR activities and the LPS-induced IL-1β secretion, and/or restored the compromised geranylgeranylation of GTPases (20, 51, 52, 56). While promising, supplementation of intermediate isoprenoids so far has not been studied in MKD patients. In particular the dietary supplementation of GGPP or its precursor geranylgeraniol could be interesting as potential treatment to prevent the inflammatory episodes in MKD patients. A recent study reported that a diet with either beef or soybean, which both contain high levels of GGPP, resulted in increased plasma levels of GGPP in healthy subjects (111). Moreover, in patients with dyslipidaemia a diet high in GGPP reversed both the statin-induced decrease of plasma GGPP levels and the RhoA activity in blood monocytes (111). It should be noted, however, that little is known about possible adverse effects of a high intake of GGPP or geranylgeraniol. A toxicological study with annatto oil, which contains approximately 80% geranylgeraniol did not reveal mutagenic or genotoxic effects. However, in a 90-day study in rats, the intake of annotto oil resulted in irritation of the fore-stomach with all doses tested, and intermediate and high doses resulted in adverse liver problems (112).

Perhaps the most promising therapeutic approach would be the inhibition of squalene synthase, which is the first enzyme in the isoprenoid biosynthesis pathway committed to the synthesis of sterol isoprenoids (**Figure 1**). Inhibitors of squalene synthase have been developed for lowering cholesterol levels as an alternative to statins, and have been tested in clinical trials, but are currently not used in patients for this purpose. Similar to statins, the squalene synthase inhibitors lower intracellular cholesterol levels, which activates SREBP-2, leading to increased expression of genes encoding the enzymes of the isoprenoid biosynthesis pathway. However, in contrast to statins, which inhibit the synthesis of all isoprenoids, inhibition of squalene synthase only inhibits the synthesis of the sterol isoprenoids and thus redirects the flux of the isoprenoid biosynthesis pathway towards the synthesis of non-sterol isoprenoids. Accordingly, incubation of MKD fibroblasts with the fungal metabolite and squalene synthase inhibitor zaragozic acid A resulted in increased MK enzyme activity as a consequence of increased *MVK* gene transcription, and restored the compromised geranylgeranylation of RhoA and Rac1 (113). Moreover, zaragozic acid A reversed the LPS-induced IL-1β release in HIDS patients’ derived PBMCs (52). In a later study, TAK-475 M-I, the active metabolite of another squalene synthase inhibitor lapaquistat acetate (TAK-475), was shown to reduce the LPS-induced IL-1β release in simvastatin-treated PBMCs and THP-1 cells (114). So far, TAK-475 is the only squalene synthase inhibitor that reached phase 2 and 3 clinical trials, in which it was tested as potential anti-hyperlipidemic drug. However, in spite of several favorable properties, further development of TAK-475 was discontinued, as a high dose of TAK-475 resulted in signs of hepatotoxicity, whereas the effects of a lower dose were not superior to already approved lipid-lowering therapies (115).

## Other Disease Phenotypes Related to MKD

Over the last years, variants in *MVK* have been linked to disease phenotypes other than the HIDS or MA phenotype of MKD. One of these is porokeratosis (MIM #175900), a heterogeneous group of rare keratinization disorders characterized by skin lesions. Initially, heterozygous variants in *MVK* had been reported to cause disseminated superficial actinic porokeratosis (DSAP), the most common subtype of porokeratosis (116). This was followed by heterozygous variants in additional genes encoding the three enzymes following MK in the isoprenoid biosynthesis pathway, phosphomevalonate kinase, mevalonate diphosphate decarboxylase and farnesyl diphosphate synthase (117–120). This was remarkable, because porokeratosis had never been observed in heterozygous parents and siblings of known MKD patients. Moreover, although MKD patients can present with skin rash during febrile episodes, this is not comparable to the skin lesions in porokeratosis, which also start to develop later in life, usually during adulthood. More recently, however, second hit variants in *MVD* or *MVK*, the genes respectively encoding mevalonate diphosphate decarboxylase and MK, were identified in affected skin lesions. This indicated that, in the presence of a mono-allelic germline variant a second hit variant can trigger the development of porokeratosis (121). These findings suggest that a shortage of (some) isoprenoids may cause the development of porokeratosis. MK was found to protect against type A ultraviolet induced apoptosis and affected the regulation of calcium-induced differentiation of keratinocytes (116). *MVK* shRNA experiments in a keratinocyte cell line decreased expression of differentiation markers, protein prenylation and increased apoptosis. These effects were reversed upon addition of FPP or GGPP to the cells (122).

Variants in *MVK* have also been linked to retinitis pigmentosa (RP) and early onset inflammatory bowel disease (IBD). RP is an inherited form of retinal dystrophy, characterized by night blindness and peripheral vision loss. There is large variation in the genetic background and the clinical manifestation of RP (123). Multiple case series found biallelic variants in the *MVK* gene in patients with RP, which is less surprising given the fact that ocular involvement, including RP, already had been associated with MKD. Upon clinical re-evaluation, these patients not only showed symptoms of RP but also a variety of symptoms reported for MKD, ranging from HIDS symptoms during childhood to more severe symptoms related to the MA phenotype (11, 124–127).

Finally, MKD is one of the monogenetic diseases that can present with severe and early-onset IBD-related symptoms (128). Genetic overlap between MKD and IBD was found in six genetically confirmed MKD patients, who all experienced abdominal pain and frequent episodes of diarrhea in their first year of life, and who also carried genetic variants associated with the development of IBD (129).

## Discussion

**Figure 3:**
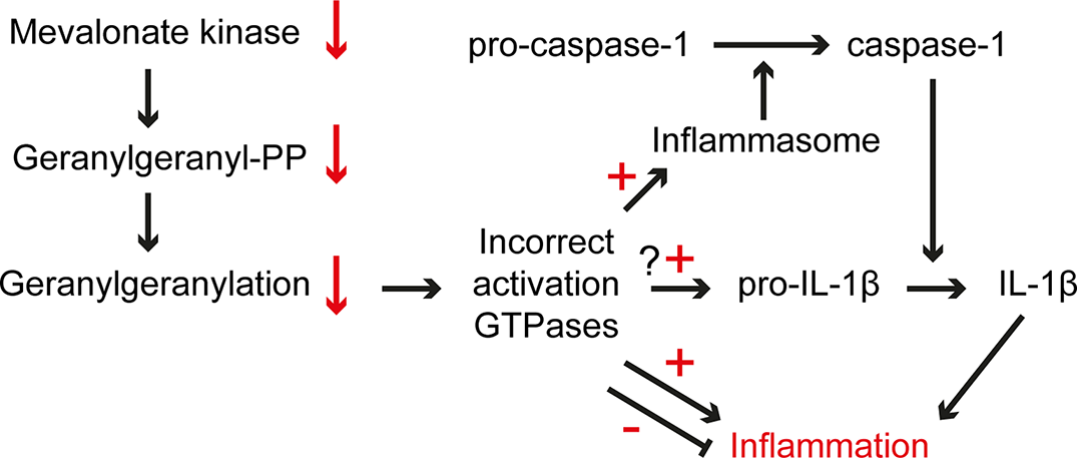
Pathogenic mechanisms in MKD. The low residual MK activity in MKD cells is sensitive to increased temperatures, which causes an instant further lowering of the activity and consequently a decreased flux in isoprenoid biosynthesis, which affects the generation of GGPP. The lack of GGPP affects protein geranylgeranylation resulting in ectopic activation of small GTPase, among which GTPases involved in the regulation of the innate immune response. This may cause uncontrolled activation and/or dampening of an inflammatory response.

The episodic inflammatory symptoms associated with the decreased enzyme activity of the isoprenoid biosynthetic enzyme MK in MKD underline an important role for isoprenoids in the regulation of the innate immune response. The inflammation in MKD does not appear as a direct consequence of the decreased MK activity itself, but due to the inability to respond rapidly to an instant further decrease in the temperature-sensitive activity of MK. Indeed, despite the decreased MK activities, MKD patients can still generate sufficient isoprenoid end products under normal circumstances, due to an increased activity of HMGR in patients’ cells which compensates for the decreased flux of the isoprenoid biosynthesis pathway. The rapid decrease in MK activity, e.g. due to an increased body temperature, causes a block in the isoprenoid biosynthesis with as predominant effect a (temporary) shortage of GGPP affecting one or more GGPP-dependent factors/processes required to repress a massive inflammatory response (**Figure 3**).

One of the GGPP-dependent processes that is disturbed in MKD is the prenylation of small GTPases. It is remarkable that although the temporary GGPP deficiency in MKD appears to affect different classes of prenylated proteins, including many GTPases involved in different signaling pathways, it primarily results in a pro-inflammatory phenotype characterized by transient inflammatory episodes. This may be due to the transient nature of the block in GGPP synthesis combined with a rapid response of the innate immune defense system, while other possible consequences may require longer lasting defective signaling. In the more severe MA phenotype, however, additional clinical consequences are observed, many of them already occurring prenatally.

A disturbed isoprenoid biosynthesis and in particular the shortage of GGPP has opposite consequences for the expression of pro- and anti-inflammatory cytokines. The finding that depletion of GGPP causes an increase in the pro-inflammatory IL-1β and a decrease in the anti-inflammatory IL-10 may form an explanation of the hyper-inflammatory nature of the inflammatory episodes in MKD. This also highlights that a shortage of GGPP has cell-specific consequences. Moreover, the fact that GTPases are part of a complex regulatory network, with overlapping functions and regulation, makes it challenging to identify which (combination of) prenylated proteins contribute to a pro-inflammatory phenotype in the case of GGPP shortage. Nevertheless, a direct link was found between loss of geranylgeranylation of two small GTPases and activation of the pyrin inflammasome (71, 72), confirming the early postulation that loss of prenylation is one of the important causes leading to inflammation in MKD (16, 20).

The identification of additional disease manifestations caused by MK deficiency not only enlarges the disease spectrum, but also underlines the idea that a shortage of one or more isoprenoids leads to inflammation. Moreover, the finding that mevalonate induces trained immunity *ex vivo* and that monocytes of HIDS patients have a trained immunity phenotype, shows that also other isoprenoids and mechanisms might contribute to the pro-inflammatory phenotype of MKD. Despite increased understanding of the pathophysiology of MKD, it is still unclear what exactly triggers an inflammatory episode and which mechanisms subsequently lead to inflammation *in vivo.*

Current treatment of MKD patients is mainly focused on the suppression of inflammatory symptoms. However, an interesting alternative may be to target the isoprenoid biosynthesis pathway itself, e.g. by supplementation of geranylgeraniol as precursor of GGPP, or by using squalene synthase inhibitors. These inhibitors only inhibit the synthesis of the sterol isoprenoids, thus preventing the sterol-regulated transcriptional down-regulation of isoprenoid biosynthetic genes and redirecting the flux of the pathway towards the synthesis of non-sterol isoprenoids, including GGPP. This results in increased MK activity, which potentially makes cells less sensitive to i) a sudden drop in MK activity, ii) a subsequent decrease of the flux through the pathway, and iii) the reduced synthesis of GGPP. Results from pre-clinical studies underline the potential of squalene synthase inhibitors in the treatment of MKD, which recently was also highlighted by Marcuzzi et al. for TAK-475 (130).

In summary, studying the underlying mechanisms of the inflammatory episodes that are characteristic for MKD provides increasing insight in the role of isoprenoids, and specifically of compromised protein prenylation, in the regulation of inflammation.

## Author Contributions

FP and HW conceived and wrote the manuscript. All authors contributed to the article and approved the submitted version.

## Funding

This work was funded in part by a grant from the AMC foundation.

## Conflict of Interest

The authors declare that the research was conducted in the absence of any commercial or financial relationships that could be construed as a potential conflict of interest.

## Publisher’s Note

All claims expressed in this article are solely those of the authors and do not necessarily represent those of their affiliated organizations, or those of the publisher, the editors and the reviewers. Any product that may be evaluated in this article, or claim that may be made by its manufacturer, is not guaranteed or endorsed by the publisher.
